# Elevated levels of the stress hormone, corticosterone, cause ‘pessimistic’ judgment bias in broiler chickens

**DOI:** 10.1038/s41598-017-07040-y

**Published:** 2017-07-31

**Authors:** Oluwaseun S. Iyasere, Andrew P. Beard, Jonathan H. Guy, Melissa Bateson

**Affiliations:** 1 School of Agriculture, Food and Rural Development, Newcastle University, Newcastle upon Tyne, NE1 7RU UK; 2 Institute of Neuroscience and Centre for Behaviour and Evolution, Newcastle University, Framlington Place, Newcastle upon Tyne, NE2 4HH UK; 3 Department of Animal Physiology, Federal University of Agriculture, Abeokuta, Ogun State Nigeria P.M.B 2240

## Abstract

Pessimistic judgment biases, whereby humans or non-human animals interpret ambiguous information negatively, are hypothesised to be one of the suite of adaptive changes that comprise the vertebrate stress response. To test this hypothesis, we asked whether experimentally elevating levels of the glucocorticoid stress hormone, corticosterone, in broiler chickens produced a pessimistic judgment bias. We trained young chickens to discriminate a stimulus (paper cone) placed at two locations in an arena, one associated with reward (mealworms) and one with punishment (air puff). During seven days of non-invasive administration of either corticosterone or vehicle control, we tested the birds’ responses to the cone placed at ambiguous locations between the trained locations. Corticosterone-treated birds were more likely than controls to respond as if punishment was likely when the cone was placed near to the punished location. The degree of this ‘pessimism’ was associated with smaller relative spleen weight, which is a documented consequence of chronic stress in chickens. We conclude that changes in corticosterone levels in chickens are sufficient to cause a specific change in decision making, dubbed ‘pessimism’, whereby corticosterone-treated birds showed an increased expectation of punishment in the face of ambiguous information. Pessimism could be a useful welfare indicator in chickens.

## Introduction

Pessimistic judgment biases occur when subjects interpret neutral or ambiguous information negatively, displaying either an increased expectation of punishment and/or a decreased expectation of reward in response to ambiguous cues^1^. Pessimism of these types is a common symptom of human affective disorders such as anxiety and depression^2, 3^. It has also been reported in a range of non-human animal species exposed to various different stressors^4–6^. Since stress has a role in the development, maintenance and re-emergence of affective disorders^7^, it is a plausible hypothesis that the physiological stress response is involved in the mechanism underlying the induction of pessimistic judgment biases.

In vertebrate animals, exposure to stressors triggers a highly conserved series of neuroendocrine responses throughout the body. This involves an immediate (0–20 minutes) response orchestrated by catecholamine neurotransmitters (e.g. noradrenaline and dopamine) and a medium-term (3–120+ minutes) response orchestrated by non-genomic and subsequently genomic effects of corticosteroid hormones (predominantly cortisol in humans but corticosterone in rodents and birds; henceforth both referred to as CORT)^8, 9^. The function of these responses is to facilitate rapid detection of threats, to bring about an adaptive response to present threats and to prepare the organism for future threats. According to a recent model^10^, two large-scale neurocognitive networks within the brain, namely the salience network and the executive control network, are regulated by exposure to acute stress in a time-dependent and reciprocal fashion. The early stage of the stress response, when both catecholamines and corticosteroids are elevated, is associated with a rise in salience network activity at a cost of executive control network activity. Whereas, the later stage of the stress response, when only corticosteroids remain elevated, is associated with a reversal of this balance^10^. From a functional perspective, the early phase of the stress response produces an immediate, hyper-vigilant state accompanied by an increased emphasis on rapid, automatic, stimulus response behaviour designed to deal efficiently with current threat. Whereas the later phase produces an emphasis on slower, higher-order cognition once the immediate threat has receded and the focus shifts to updating cognitive strategies for dealing with future challenges.

Evidence for potentially adaptive changes in cognition in the later stages of the stress response currently comes from studies of working memory. In both humans^11^ and rats^12^, improvements in working memory performance have been documented four hours after exposure to acute stress (CORT administration in humans and a forced swim paradigm in rats). In the current paper we seek to extend the exploration of how stress changes cognition to the study of decision making and specifically judgment bias. We predict that a change in judgment bias, whereby an animal displays an increased expectation of threat (punishment) in the face of ambiguous cues, could be one of the adaptive cognitive changes that occurs in the secondary, corticosteroid-mediated phase of the stress response, when executive control predominates. It makes evolutionary sense for an animal that has recently been exposed to a threat to alter its threshold for responding to ambiguous cues of possible future threat, because events of biological significance are often auto-correlated in time and space^9, 13^. Thus we hypothesise that repeated or chronic exposure to stress should cause a shift towards increased expectation of threat in the face of ambiguous cues. Testing this hypothesis requires showing that selective, experimental manipulation of the neuroendocrine changes that characterise the stress response causes the predicted changes in judgment bias.

Judgment bias in animals can be measured via a range of analogous behavioural tasks collectively referred to as judgment or cognitive bias tasks. In a typical judgment bias task, subjects are initially trained to associate one stimulus—positive—with a high-valued reward and another stimulus—negative—with either punishment or lack of reward. Once the subjects have acquired the discrimination between the positive and negative stimuli, they are subsequently tested by presenting them with novel, ambiguous stimuli intermediate between the two trained stimuli. Ambiguous stimuli are presented at low frequency and are neither rewarded nor punished to reduce the likelihood that the animals will learn about these stimuli during the course of the test^14, 15^. In the test trials, animals that respond to the ambiguous stimuli as if they were the positive stimulus are interpreted as displaying a high expectation of reward in the presence of ambiguous information, and hence an ‘optimistic’ cognitive style indicative of a positive mood. In contrast, animals that respond to the ambiguous stimuli as if they were the negative stimulus are interpreted as displaying a higher expectation of punishment or lower expectation of reward, and hence a more ‘pessimistic’ cognitive style indicative of a more negative mood.

One previous study has explored whether direct experimental pharmacological simulation of the stress response produces changes in interpretation of ambiguous cues in a judgment bias task. Enkel *et al*.^16^ trained rats on an active-choice judgment bias task. Twelve rats were first trained to discriminate two tones differing in frequency. On hearing the positive tone (2 or 9 kHz; counterbalanced across individuals) rats were required to press a positive lever to receive a reward of sweetened condensed milk, whereas on hearing the negative tone (9 or 2 kHz) they were required to press a negative lever to escape an electric shock. Once rats reached a stable baseline of correct discrimination performance, their responses to three ambiguous tones intermediate between the positive and negative tone (3, 5 and 7 kHz; named near-positive, medial and near-negative as appropriate) were tested. Ambiguous tones were presented at a low frequency, interspersed between positive and negative tones, and responses to ambiguous tones were neither reinforced nor punished. The rats were tested in two drug conditions, treatment and control, delivered to all subjects in a cross-over design. The treatment comprised a cocktail of CORT (0.5 mg/kg delivered via intraperitoneal injection 30 mins prior to testing) + Reboxetine (a noradrenaline reuptake inhibitor; 15 mg/kg delivered via gastric gavage 60 min prior to testing) chosen to mimic the early stages of an acute stress response when both CORT and noradrenaline are elevated. The control comprised saline delivered via the same routes as the treatment. Rats received three consecutive days of testing in each condition separated by 4 weeks of washout and discrimination retraining. The CORT + Reboxetine treatment reduced responding to the positive lever for all tones (including positive and negative), increased response omissions to positive, near-positive and medial tones and had no significant effect on responding to the negative lever. Together, these effects resulted in an overall increase in the proportion of negative judgments in the CORT + Reboxetine treatment condition compared to the control condition. This result was interpreted by the authors as showing the predicted switch towards more negative (i.e. pessimistic) judgment bias when stressed^16^.

Despite Enkel *et al*.’s^16^ apparently confirmatory result, there are potential problems with their experiment that cloud the interpretation of the data. Due to the relatively poor performance of the rats in the basic discrimination task—a criterion of only 60% correct avoidance was required for the negative tone—the rats would have experienced regular electric shocks throughout training with the negative tone (a mean of 25 days) and judgment bias testing (6 days). Furthermore, despite the claims of the authors that the rats were well habituated to the procedures, the i.p. injections and gastric gavage in the control condition were unlikely to have been without stress. Therefore, all the rats were likely to have been both chronically and acutely stressed from repeated exposure to shocks, injections and gavage at the time of the judgment bias tests, irrespective of the experimental treatment they were currently receiving. Thus, although a treatment difference in judgment bias was reported, it is not clear that this corresponds to a difference between stressed and non-stressed animals, since endogenous levels of both CORT and noradrenaline are likely to have been high in both experimental groups. This is important because the effects of stress are acknowledged to be non-monotonic, meaning that the absolute levels of stress in the two groups of rats is likely to be important in determining how they behave^17^. A second problem concerns the interpretation of the results obtained. The overall increase in the proportion of negative judgments reported in the treatment condition was driven by a reduction in the number of positive judgments. There was no significant change in the absolute number of negative judgments as would have been predicted by the evolutionary model described above^9, 13^. Furthermore, there was no suggestion that the treatment effects were restricted to the ambiguous tones, but instead appeared equal for trained and ambiguous tones, again at odds with the evolutionary model. Thus in summary, this experiment does not provide strong support for our hypothesis that the neuroendocrine changes associated with acute stress cause a pessimistic judgment bias characterised by an increased expectation of threat (punishment) in the face of future ambiguous information.

The aim of the current experiment was to extend the exploration of the effects of CORT on judgment bias addressing the problems highlighted above with Enkel *et al*.’s study. Specifically, we developed a judgment bias task involving punishment that was faster to learn and therefore did not itself impose chronic stress on the experimental animals. Furthermore, we used a non-invasive method of CORT administration in order to avoid acute stress associated with the administration route in our control group. Via the use of these innovations we tested the hypothesis that elevated levels of CORT, typical of a period of chronic stress, cause pessimistic judgment biases characterised by an increased expectation of punishment in the face of an ambiguous cue. The study was conducted in broiler chickens (*Gallus gallus domesticus*), an economically important species given its dominance in global meat production for which a behavioural measure of stress would be useful in assessing the welfare impact of different husbandry regimes.

**Figure 1:**
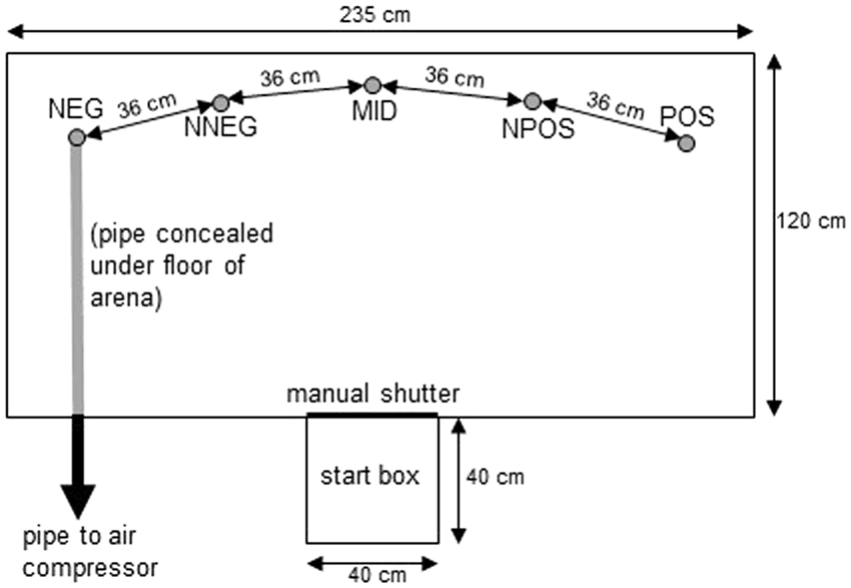
Plan of the arena used for training and testing (not to scale). The circles show the five positions in which cones could be placed during the judgment bias task namely: NEG (negative; punished with an air puff), NNEG (near-negative; ambiguous closest to air puff), MID (middle; ambiguous equidistant from air puff and reward), NPOS (near-positive; ambiguous closest to reward) and POS (positive; rewarded with a mealworm). Note that on a given trial only a single cone was presented. The cones were made from a 3-cm diameter circle of paper cut to the centre and taped into a cone, presented on an 8-cm diameter petri dish.

The judgment bias task that we developed was based on the spatial, go/no-go judgment bias task originally developed for rats by Burman *et al*.^18^ and subsequently adapted for chickens^19, 20^. On each trial an individual chicken was released into an arena (Fig. 1) in which we had placed a petri dish containing a single white paper cone, that could be used to conceal either palatable mealworms, or the orifice from which an aversive air puff emanated (air puffs have previously been shown to be aversive to chickens^21, 22^) or nothing. The bird was required to make a decision about whether to approach and displace the cone within a designated time limit. The spatial location of the cone provided a discriminative stimulus indicating the outcome of displacing the cone. Birds were initially trained to discriminate two locations: POS was associated with reward with two mealworms hidden under the cone and NEG was associated with an aversive air puff emanating from the location of the cone. Following acquisition of this discrimination, birds were randomly allocated to two treatment groups: corticosterone (CORT) and control. For the next seven days the birds were fed treated mealworms (either CORT or control) in order to non-invasively experimentally manipulate basal CORT levels. On the third day after the start of this manipulation, both groups were tested with cones placed in three ambiguous locations intermediate between the two trained locations, namely near-POS (NPOS), middle (MID) and near-NEG (NNEG; as shown in Fig. 1). These test cones were presented in extinction (i.e. with nothing underneath them). We used the latency of the bird to approach the cone as a measure of its pessimism regarding the likely outcome, with greater latencies indicating a higher expectation of punishment with an air puff. Behavioural testing continued for a total of three days. At the end of the experiment the birds were euthanized in order to examine the anatomical effects of the CORT treatment.

## Results

### Discrimination training

A total of 17 birds completed discrimination training (10 from replicate 1 and 7 from replicate 2). Of these, 15 birds met the criteria for showing a significant discrimination between the POS and NEG locations (two-tailed, Wilcoxon, two-sample tests, all P < 0.05). These 15 birds approached and displaced 23.60 ± 1.45 (mean ± sd) of the 24 POS cones presented during the four days of discrimination training, but only 4.13 ± 4.36 of the 24 NEG cones presented. Thus, the birds learned fast and each trained bird received a mean of only 4.13 air puffs during discrimination training. Of the 15 successfully trained birds, 14 were randomly selected and randomly allocated to treatment groups (7 CORT and 7 control). In each experimental replicate equal numbers of birds were allocated to each treatment group in order to create a fully balanced experimental design. These 14 birds progressed to judgment bias testing.

### Judgment bias testing

To test whether birds retained their discrimination of the POS and NEG locations after the CORT build-up phase and during the three days of judgment bias testing, we compared the latencies from the nine POS and nine NEG trials pooled over the three days of testing. All 14 birds remained significantly faster to probe POS than NEG stimuli (Table 1: all P < 0.05) and were hence retained in the subsequent analyses of the judgment bias data.

**Table 1:** Difference in latencies to approach POS and NEG during judgment bias testing.

Bird ID	Treatment	POS latency (mean ± SE)	NEG latency (mean ± SE)	Wilcoxon two-sample test statistic^1^	P-value^2^
8	Control	11.00 ± 2.52	60.00 ± 0	81.0	<0.001*
10	Control	3.56 ± 0.29	43.78 ± 7.36	78.5	<0.001*
15	Control	17.44 ± 6.09	60.00 ± 0	76.5	<0.001*
16	Control	23.11 ± 9.22	43.89 ± 5.96	60.0	0.040*
25	Control	3.78 ± 0.22	14.56 ± 4.18	81.0	<0.001*
31	Control	6.11 ± 0.35	60.00 ± 0	81.00	<0.001*
12Y	Control	8.67 ± 5.65	52.89 ± 5.11	79.0	<0.001*
2	CORT	17.78 ± 4.97	60.00 ± 0	81.0	<0.001*
7	CORT	43.33 ± 8.39	60.00 ± 0	54.0	0.039*
14	CORT	30.56 ± 9.38	54.44 ± 5.56	60.0	0.028*
18	CORT	3.78 ± 0.04	30.00 ± 7.88	81.0	<0.001*
20	CORT	3.22 ± 0.15	60.00 ± 0	81.0	<0.001*
30	CORT	8.00 ± 1.31	60.00 ± 0	81.0	<0.001*
12B	CORT	16.22 ± 5.65	60.00 ± 0	76.5	<0.001*

Notes: ^1^For all birds N_POS_ = N_NEG_ = 9; ^2^P-values are from a one-sided test of the alternative hypothesis that the POS latency is shorter than the NEG latency; *P < 0.05.

**Figure 2:**
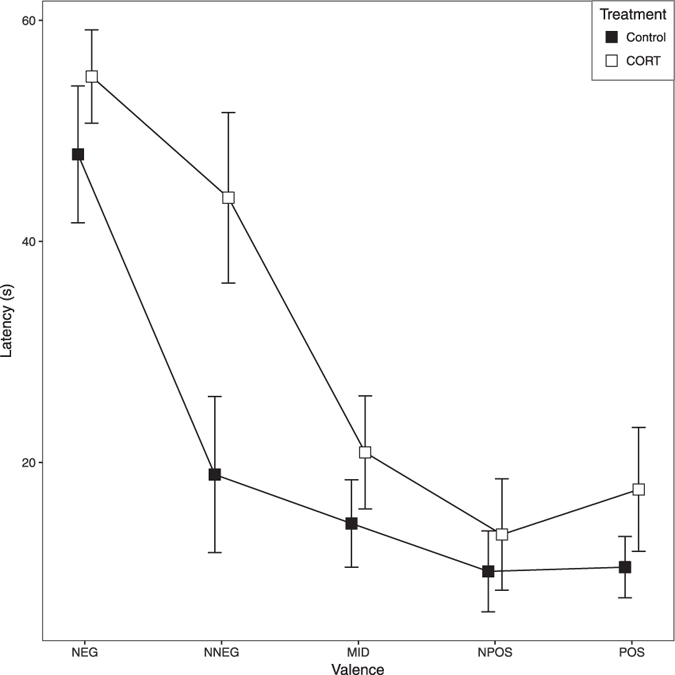
Birds treated with corticosterone (CORT) were more pessimistic than control birds in trials in which the cone was placed at the near-negative location (NNEG). Line graph showing the mean ± 1 SEM latencies of the birds in the two treatment groups to approach the cone at each of the five locations in the judgment bias test. The significant valence x treatment interaction (see text for details) occurs because the CORT birds were slower to approach the NNEG cone than the control birds.

Figure 2 shows that birds in the control treatment were faster to displace cones at all locations than birds in the CORT treatment. An effect of CORT on latency at the POS and NEG locations is interesting in its own right, but also complicates interpretation of the cognitive bias data. To test whether this difference was significant we fitted a GLMM with latency to displace cones at POS and NEG locations as the dependent variable and the following fixed predictors: valence (categorical: 1 = NEG or 5 = POS), treatment (categorical: CORT or control) and the valence by treatment interaction. As expected based on the results in Table 1 there was a significant main effect of valence on latency, with birds being faster to displace cones in the POS location than in the NEG location (GLMM: B ± SE = −37.35 ± 2.71, LR = 140.53, P < 0.001). Although CORT birds were slower than control birds, the main effect of treatment was not significant (GLMM: B ± SE = 7.05 ± 5.51, LR = 1.77, P = 0.183), and neither was the valence by treatment interaction (GLMM: B ± SE = −0.02 ± 3.84, LR < 0.01, P = 0.997). To test whether there was significant variation in latency between individual birds, we compared the above model with and without the random effect of bird. This comparison showed that there were significant differences between birds in latency to displace POS and NEG cones (LR = 47.42, P < 0.001). Based on the above findings we made the decision to control for individual differences in latency to displace POS and NEG cones in our subsequent analysis of the birds’ responses to cones placed at ambiguous locations (see refs 23 and 24 for a similar analysis approach in previous cognitive bias studies).

As a measure of each bird’s overall speed to displace cones, we calculated its mean latency to displace cones in the POS and NEG locations over the 3 days of testing; henceforth this variable is called ‘speed’. By including speed as a covariate in the subsequent analysis of the latencies to displace cones in ambiguous locations, we controlled statistically for individual differences in speed. To explore whether treatment affected the birds’ response to the ambiguous stimuli (NPOS, MID and NNEG), we fitted a model with latency to displace cones in ambiguous locations as the dependent variable and the following fixed predictors: speed (continuous), valence (continuous variable designating the ambiguity: 2–4; where 2 = NNEG, 3 = MID and 4 = NPOS), treatment (categorical: CORT or control) and the valence by treatment interaction (we experimented with adding test day as an additional predictor, but since the effect of day was not significant, and it had no effect on the significance of other variables in the model, we excluded it from the final model presented). There was a significant effect of speed on latency to displace cones at ambiguous locations, with birds that were faster at POS and NEG also being faster at the ambiguous locations (GLMM: B ± SE = 0.77 ± 0.20, LR = 10.16, P = 0.001). There was also a significant main effect of valence on latency to probe the ambiguous stimuli, with birds being faster to displace cones at locations more similar to POS (GLMM: B ± SE = −4.38 ± 2.90, LR = 20.40, P < 0.001). The main effect of treatment was not significant (GLMM: B ± SE = 38.76 ± 12.98, LR = 2.14, P = 0.144), but there was a significant interaction between valence and treatment (GLMM: B ± SE = −10.85 ± 4.10, LR = 7.07, P = 0.008). This interaction occurred because birds in the CORT group were slower to displace cones located at the NNEG location than birds in the control group (see Fig. 2).

### Anatomy and blood physiology

The effects of the CORT treatment on anatomy and blood physiology are summarised in Table 2. In testing for significant differences between the CORT and control treatments we assumed a criterion for significance of P ≤ 0.006 (the Bonferroni-corrected criterion for significance with eight tests). After controlling statistically for individual differences in body weight on day 17, CORT birds had significantly heavier livers and lighter spleens than control birds. No other significant differences in anatomy or blood physiology were found.

**Table 2:** Effects of the experimental treatment on anatomy and blood physiology.

Measure^1^	Control			CORT			Inferential statistics		
	N	Mean	SE	N	Mean	SE	Test statistic and df^2^	P-value	Significance
Weight gain (g)	10	326.80	15.02	12	314.42	23.03	t(18.3) = 0.45	0.658	
Heart (g)	11	8.11	0.30	12	8.34	0.45	F(1,20) = 0.012	0.912	
Liver (g)	11	46.05	3.10	12	62.08	3.20	F(1,20) = 39.00	<0.001	*
Spleen (g)	11	1.88	0.16	12	1.48	0.12	F(1,20) = 9.37	0.006	*
Blood pH	7	7.36	0.04	9	7.41	0.02	t(8.57) = −0.96	0.362	
Blood pCO_2_ (mmHg)	7	41.11	4.51	9	39.10	1.49	t(7.32) = 0.42	0.684	
Blood Na^+^ (mmol/l)	7	145.43	0.69	9	143.78	0.22	t(7.27) = 2.29	0.054	
Blood glucose (ng/dl)	7	237.71	3.94	9	266.78	12.97	t(9.44) = −2.14	0.059	

Notes: ^1^With the exception of Weight gain, which was measured between days 11 and 17, all measures were taken at the end of the experiment on day 17. ^2^For Weight gain, pH, pCO2, Na^+^ and Glucose the test was a two-sample t-test; for Heart, Liver and Spleen, the test was an ANCOVA with weight on day 17 included as the covariate. *Indicates a P-value ≤ 0.006.

### Correlations between anatomical changes and behavioural data

**Figure 3:**
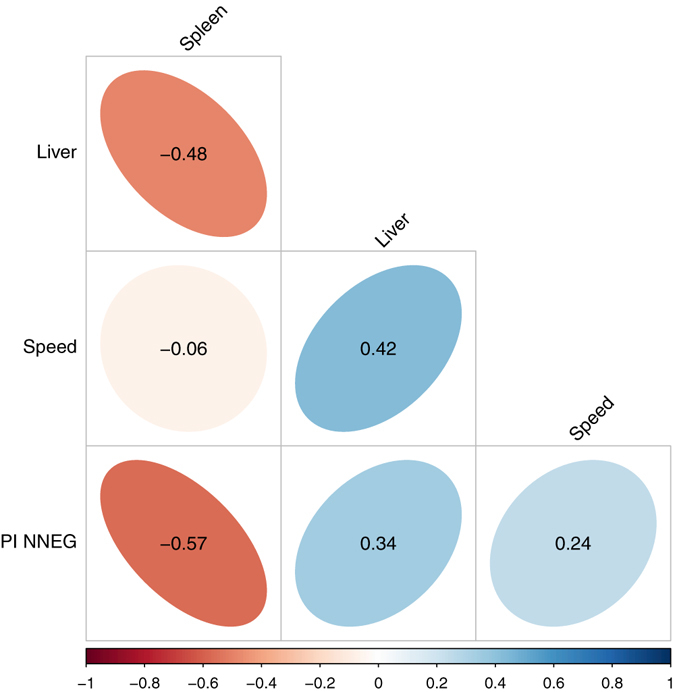
Relative spleen size is negatively correlated with pessimism. Visualisation of the Pearson correlations between: relative liver weight, relative spleen weight, speed (i.e. mean latency to displace cones at POS and NEG locations in the cognitive bias test) and the pessimism index for the NNEG trials (PI NNEG; see text for details). The shape and colour of the ellipses represent the direction and strength of the correlations; Pearson correlation coefficients are given in the centre of each ellipse. The critical value of *r* for significance (at P < 0.05) in a two-tailed Pearson correlation with 12 df is equal to ±0.53. Thus, the only significant correlation is that between relative spleen weight and the Pessimism Index.

On the assumption that relative liver weight and relative spleen weight might provide continuous proxy measures of the degree of stress induced by the CORT treatment in each bird, we next explored how these measures correlated with the behavioural data from the cognitive bias test. Our hypothesis was that if the magnitude of pessimistic cognitive bias provides a behavioural read-out of the negative affective state arising from the CORT treatment, then we should expect correlations between the anatomical and behavioural data. Relative organ weights for each bird were obtained by dividing organ weight by body weight on day 17. A pessimism index for each bird was obtained using the following formula: Pessimism Index = (mean latency to NNEG–mean latency to POS)/(mean latency to NEG – mean latency to POS). This index is equal to 0 if the bird responds to NNEG identically to POS, and to 1 if the bird responds to NNEG identically to NEG. Thus, a larger number indicates greater pessimism. Figure 3 shows a matrix of Pearson correlation coefficients between relative liver weight, relative spleen weight, speed (i.e. mean speed to POS and NEG over the three days of testing) and the Pessimism Index. In line with our hypothesis, birds with relatively smaller spleens were significantly more pessimistic (Pearson correlation: r(12) = −0.57, P = 0.034). No other significant correlations were found.

**Figure 4:**
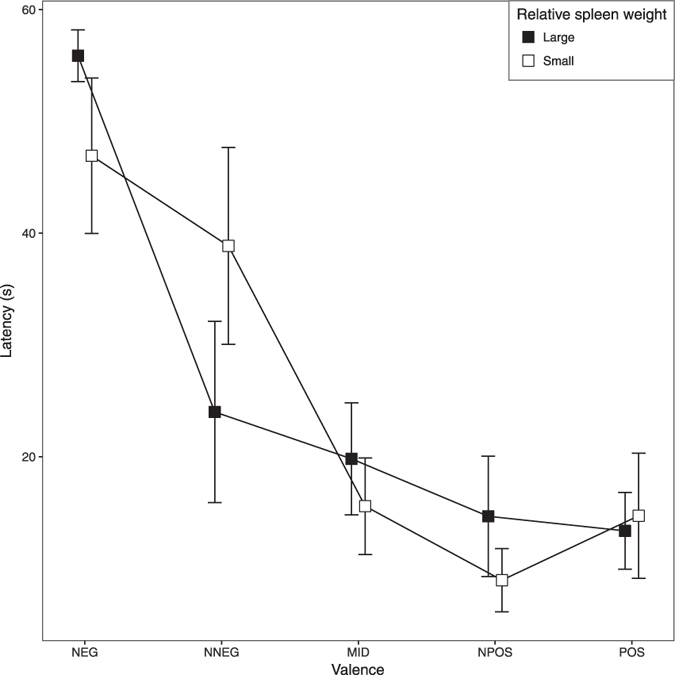
Birds with relatively smaller spleens are more pessimistic in trials in which the cone was placed at the near-negative location (NNEG). Line graph showing the mean ± 1 SEM latencies of the birds in two relative spleen size groups to approach the cone at each of the five locations in the judgment bias test. The two spleen size groups were obtained via a median split of the data; note that this is for visualisation purposes only, and the statistical model reported in the text modelled relative spleen size as a continuous variable. The significant valence x relatively spleen size interaction (see text for details) occurs because the birds with relatively small spleens were slower to approach the NNEG cone.

If relative spleen weight is a proxy measure of chronic CORT exposure, we hypothesised that relative spleen weight should predict performance on the judgment bias task, and furthermore, that it might be a better predictor of behaviour on the task than the CORT treatment group itself. To explore whether relative spleen weight predicted the birds’ response to the ambiguous stimuli (NPOS, MID and NNEG) in the judgment bias task better than treatment, we repeated the analysis presented previously replacing treatment with relative spleen weight. As previously, there were significant main effects of speed and valence on latency to displace cones at ambiguous locations (GLMMs for speed: B ± SE = 0.85 ± 0.18, LR = 13.45, P < 0.001; and for valence: B ± SE = −34.28 ± 8.31, LR = 20.41, P < 0.001). The main effect of relative spleen weight was marginally non-significant (GLMM: B ± SE = −84846.63 ± 24476.89, LR = 3.38, P = 0.066). However, there was a significant interaction between valence and relative spleen weight (GLMM: B ± SE = 23697.14 ± 7800.33, LR = 9.22, P = 0.002). This interaction occurred because birds with relatively small spleens were slower to displace cones located at the NNEG location (Fig. 4). To compare the two models we used the R package AICcmodavg^25^ and a modified version of Akaiki’s Information Criterion (AICc) recommended for small sample sizes^26^. The model with relative spleen size as a predictor provided a better fit to the data than the previous model with CORT treatment: AICcs were 1107.49 and 1110.88 for the spleen weight and CORT models respectively giving a ΔAICc of 3.39 (a reduction in AICc of at least 2 units is generally regarded as significant^26^).

## Discussion

Our aims were to develop a judgment bias task for chickens involving punishment that did not in itself impose chronic stress on the birds, and to use this task to test the effects of non-invasively-ingested corticosterone (CORT) on decision making. We sought to test the hypothesis that CORT-fed birds with elevated basal CORT levels would show enhanced anticipation of punishment in the face of ambiguous stimuli. Our data clearly support this hypothesis. Using a spatial judgment bias task in which birds were trained to discriminate a white paper cone placed at two locations, one associated with reward (mealworms) and one with punishment (an aversive air puff), we tested the effects of CORT on birds’ responses to cones placed at ambiguous locations using a between-subjects design. CORT-fed birds were more likely than control birds to respond ‘pessimistically’ in the test trials, approaching the cone more slowly or not at all. Importantly, this pessimism was restricted to test trials in which the ambiguous cone was placed near to the punished location (NNEG), suggesting that it was caused by an increased expectation of punishment as opposed to a decreased expectation of reward.

Our results from the judgment bias task are similar to those obtained in rats by Enkel *et al*.^16^, in that both experiments showed an increase in pessimistic judgment bias as a result of treatment with CORT. However, our results differ from those of Enkel *et al*. in the source of the pessimism: in our study, the data suggest that CORT produced an increased expectation of punishment, whereas in Enkel *et al*.’s study CORT + Reboxitine produced a reduction in positive lever presses, implying a reduction in expectation of reward. It is possible that the difference between the judgment biases in the two studies arises from the fact that the CORT treatment used in the rats mimicked an earlier phase of the stress response than that induced in the chickens due to the CORT being delivered to the rats in a cocktail with Reboxitine^10^. As discussed earlier, the different phases of the stress response have different functions^10^, and these are likely to be implemented mechanistically via different cognitive biases. The early phase of the stress response, mediated predominantly by catecholamines, functions to maximise immediate survival by supressing any unnecessary behaviour such as opportunistic foraging. Suppression of unnecessary foraging would predict reduced willingness to investigate ambiguous cues of potential reward, exactly as observed in Enkel *et al*.’s^16^ rats. In contrast, we propose that the later phase of the stress response, mediated by corticosteroids, corresponds to an anxiety-like mood state that functions, in part, to reduce exposure to further threats. This function might be implemented via increased expectation of punishment in the face of ambiguous cues of potential punishment, as observed in the chickens. Thus the subtly different judgment biases reported in the two experiments can be reconciled with current theories about the functions of the different phases of the stress response. A proper test of this hypothesis would require testing with both CORT and CORT + Reboxitine in the same experiment. Although there is growing evidence from humans suggesting that high baseline plasma CORT levels are associated with subtle biases in attention and memory that adaptively tune behaviour in the face of potential threats^11^, we believe that our study is the first in any species to show that experimentally manipulating chronic levels of CORT causes the predicted increase in expectation of punishment expected in anxiety-like mood states^9, 13, 27^.

It is a limitation of our study that we did not directly measure the plasma CORT levels in our chickens in order to prove that the CORT administration protocol worked as planned. The protocol was calculated to deliver approximately 4 mg of CORT per kg of body weight per day, a dose previously established in chickens to provide significantly elevated levels of plasma CORT, mimicking natural surges of CORT induced by environmental perturbations^28^. In a previous study, when administered to chickens via drinking water, CORT delivered at this same daily dose reached peak plasma concentrations within 24 hours^28^, suggesting that the two-day CORT build up period we used (days 11–12) should have been ample for stable plasma levels of CORT to be achieved in the CORT-fed birds. It would have been ideal to check plasma CORT levels of the birds during the experimental phase of the experiment, but we did not attempt to do this due to the difficulties of obtaining valid baseline plasma CORT measures from group-housed animals. In place of directly measuring CORT levels, we opted instead to measure more stable physiological and anatomical consequences associated with chronically elevated CORT levels. We found two significant differences between the post-mortem anatomy of CORT birds and control birds. The biggest effect was on relative liver weight, with CORT birds having significantly greater relative liver weight than control birds. The liver is the major site of fatty acid synthesis in chickens meaning that liver weight is predicted to rise in response to chronic stress. In support of this, significant differences in liver weight have been reported within 18 hours of insertion of a pump that releases ACTH in chickens^29^ (ACTH stimulates the adrenal glands to release CORT into the blood stream). Our finding mirrors that of a previous study that also found that the biggest effect of ACTH was on the size of the liver of chickens^30^. The only other significant difference that we found between the anatomy of CORT and control birds was that the CORT birds had relatively smaller spleens (an effect of CORT on top of their generally smaller body weights). Since a major function of CORT is to suppress the immune system, weights of immunological organs are predicted to reduce in response to chronic stress. In support of this, the relative weight of the spleen has been shown to be a robust effect of CORT administration in chickens^28^. Although we did not find any significant differences in the blood chemistry of CORT and control birds, blood sodium levels were marginally non-significantly lower in CORT birds and glucose levels were marginally non-significantly higher. Reduced plasma concentrations of sodium and increased plasma concentrations of glucose have both been previously reported in a chick model of stress using ACTH pump insertion for seven days^31^. Thus in summary, the differences that we found between the anatomy and physiology of the CORT and control birds are in line with those previously reported in chickens with chronically raised CORT levels. This suggests that our non-invasive CORT feeding protocol produced the differences in plasma CORT levels during the experimental phase of our study similar to the differences that can be achieved via more invasive means such as gavage, injections or pump implantation.

Interestingly, we found that the degree of behavioural ‘pessimism’ in the judgment bias task was correlated with smaller relative spleen weight. Furthermore, we showed that relative spleen weight was a better predictor of a bird’s behaviour in the judgment bias task than the CORT treatment to which it was assigned. These results are compatible with the hypothesis that the CORT treatment had slightly different effects on different birds, perhaps due to uncontrolled variation in the exact dose given and/or variation in genotype or body weight, and that the size of the effect was reflected in relative spleen size. If relative spleen size is a better measure of effective CORT dose, and CORT dose predicts pessimism on the judgment bias task, then it makes sense that relative spleen size will be a better predictor of pessimism than the CORT treatment group. However, in the absence of direct measures of CORT it is impossible to directly test this hypothesis with our data.

Cognitive bias measures are widely argued to be good measures of animal welfare^4, 32^. The rationale for this claim rests partially on evidence that cognitive biases are selectively sensitive to the valence of affective states as opposed to potentially orthogonal states such as level of arousal. In contrast, stress hormone levels are known to rise in response to both positively- and negatively-valenced states, such as for example excitement and anxiety, making them less reliable welfare indicators. In this context, it is interesting that in the current study we have experimentally manipulated stress hormone levels and shown that increased CORT is sufficient to produce a pessimistic judgment bias assumed to be indicative of negatively-valenced affective state. Our result raises the question of why the CORT feeding protocol that we used produced a pessimistic cognitive judgment bias indicative of a negative affective state? Sapolsky has argued that the CORT dose might be critical in determining the valence of its effect due to the dual receptor system for glucocoritcoids^17^. Low CORT doses (approximately 10–20 μg/dl) are hypothesised to mediate positive outcomes via high affinity, low capacity mineralocorticoid receptors, whereas higher CORT doses are hypothesised to mediate negative outcomes via low affinity, high capacity glucocorticoid receptors. If this model is correct, then by giving two CORT doses a day we were delivering intermittent high doses of CORT, mimicking the hormonal response to a negatively-valenced stressor. This interpretation could be tested by delivering mealworms containing lower doses of CORT, in which case an optimistic judgment bias, indicative of a positive affective state, would be predicted. Incidentally, Sapolsky’s model makes it clear why using integrative measures of total CORT release via faecal samples or hair may be an unreliable means of assessing animal welfare, because the same integrative dose of CORT may result in positive outcomes if it is split between many small doses spread over a period of time, but negative outcomes if it is concentrated into a fewer large doses.

A second reason for favouring cognitive biases as a welfare indicator is that in humans, optimistic cognitive biases are usually associated with subjectively positive moods, whereas pessimistic biases are usually associated with subjectively negative moods^32^. It thus appears that cognitive biases provide an objective measure of the valence of subjective experience. The possibility that non-human animals might have the capacity to subjectively experience negative affective states akin to human anxiety, depression, boredom or chronic pain is central to concerns for animal welfare. For this reason, there has been considerable effort devoted to the development of techniques for assessing the presence of such states in non-human animal species likely to be at risk of developing them. In this context, it is noteworthy that studies that have investigated the effects of single dose CORT administration on affective state in humans report an absence of effects on subjectively reported mood or emotion^33^. Whilst it is possible that repeated CORT doses might produce subjective effects in humans, these studies raise doubts about whether cognitive biases and subjective experience are necessarily as tightly linked as has previously been assumed.

We conclude that CORT is causal in the mechanistic pathway that produces pessimistic judgment bias in chickens, and that changes in CORT levels are sufficient to produce a specific change in decision making, whereby CORT-fed birds showed an increased expectation of punishment in the face of ambiguous information.

## Methods

### Ethics statement

Our study adhered to the ASAB/ABS Guidelines for the Use of Animals in Research, and was approved by the local ethical review committee at Newcastle University. It was completed under UK Home Office project licence number PPL 60/4270. All birds were judged as generally healthy prior to inclusion in the study, and were checked daily for signs of health during the study. None of the birds developed obvious lameness or poor gait during the study.

### Subjects

Experimental subjects were 42 female broiler chickens (*Gallus gallus domesticus*), of a commercial strain (Ross 308, Aviagen Ltd, Newbridge, UK). The experiment was conducted in two replicates, the second starting one week after completion of the first. Replicate one comprised 15 birds (17 days of age; average weight of 1300 g) and replicate two, 27 birds (13 days of age; average weight 950 g). Birds were housed in a laboratory (4.1 m long × 2.4 m wide × 2.2 m height) under a husbandry regimen designed to resemble commercial conditions, comprising lighting conditions of 14 L: 10D (lights on at 0800 and off at 2200), a constant temperature of 20 °C and relative humidity of 50% RH. On arrival, the birds were randomly allocated to circular pens (90 cm diameter, 30 cm height) with a maximum of 5 birds per pen. The pens were lined with wood shavings (5 cm depth). Feed (20% CP, 13.0 MJ/kg ME, 4% oil and 6% ash; W.E Jameson & Son Ltd, Masham, UK) and water were provided *ad libitum*. The birds were allowed to settle for three days. During this period they were additionally offered live mealworms (*Tenebrio molitor*; approx. 30 per pen) once a day. Since mealworms were used both to deliver the experimental manipulation, and as a reward in the cognitive bias task (see details below), it was important that all birds were familiar with this usually highly preferred food item.

### Experimental design and overview of protocol

The experiment explored the effect of a single factor (CORT treatment) with two levels (CORT and control groups) on cognitive bias using a between-subjects design. Following settling into the laboratory (days 1–3) all birds began training on the spatial discrimination necessary for the cognitive bias task (days 4–10). On day 11, a subset of 23 birds (15 that met the training criteria for the cognitive bias experiment and an additional 8 birds from replicate two kept to boost sample sizes for the blood physiology and anatomical data) were randomly allocated to one of the two experimental treatment groups and fed treated mealworms (either CORT or control) for the next 7 days (days 11–17). Days 11–12 were designated as the CORT build up period and no behavioural training or testing occurred on these days. On the third day of treatment (i.e. day 13) 14 birds that had met the cognitive bias training criteria began cognitive bias testing; testing continued for three days (days 13–15). Days 16–17 were used for additional behavioural measurements on the birds not described in the current paper. At the end of day 17, all the remaining 23 birds were blood-sampled and euthanized for organ collection.

### Arena for training and judgment bias test

Behavioural training and testing was conducted in an adjacent laboratory of identical size and environmental conditions to that used for housing the birds. The test arena (Fig. 1) consisted of an open-topped wooden box (2.35 m wide × 1.2 m long × 0.40 m high) raised off the floor on a low platform. In the centre of one of the 2.35 m walls there was an open-toped start box (0.4 m wide × 0.4 m long × 0.4 m high) separated from the main arena by a sliding door. The rewarded (POS) and punished (NEG) locations were located 1.0 m from the start box and 1.8 m apart (POS on the right of the midpoint, MID, from the bird’s perspective and NEG on the left of the MID; the arrangement was identical for all birds due to the constraints of the air puff apparatus). The ambiguous locations, used during cognitive bias testing, were also 1.0 m from the start box and were equally distributed between the POS and NEG locations. Thus MID was midway between POS and NEG, NPOS (for near-positive) was midway between POS and MID and NNEG (for near-negative) was midway between NEG and MID. At the NEG location a small hole (5 mm diameter) in the floor of the arena was connected via a pipe that ran under the floor to a portable air compressor (KNF Neuberger Ltd, Witney, UK) fitted with an air gun (Cromwell Ltd, Leicester, UK) that allowed the experimenter to deliver an air puff of mild pressure (3 bars) to the NEG location.

### Corticosterone treatment

For the 7 days of the experimental treatment (days 11–17), the CORT or control substance was administered to the birds orally by means of offering them injected mealworms to eat twice daily. This method of CORT delivery was designed to mimic natural surges of CORT induced by regular exposure to stressors. Preparation of CORT solution and injection of mealworms followed the protocol of Breuner *et al*.^34^. Birds in the CORT group were fed mealworms injected with corticosterone (Sigma-Aldrich Company, Dorset, UK) dissolved in dimethyl sulfoxide (DMSO; Sigma-Aldrich Company, Dorset, UK), a nonpolar solvent which readily dissolves crystalline corticosterone and can therefore be used for quick delivery of corticosterone into the blood stream; whereas birds in the control group were fed mealworms injected with pure DMSO. Mealworms were prepared fresh twice daily immediately prior to each feeding. They were rendered inactive by placing them on ice for about 20 mins, and then injected with either 100 mg/mL CORT or pure DMSO using a 100 μl Hamilton syringe. The aim was to provide each CORT bird with approximately 4 mg of CORT per kg of body weight per day, a dose previously established in chickens to provide significantly elevated levels of plasma CORT for up to 8 days mimicking chronic stress^28^. At the start of the treatment, birds in replicate one weighed a mean of 1500 g and those in replicate two weighed a mean of 1100 g. Hence, birds in replicate one were fed a total of 6 mg of CORT per day (two mealworms per feed, twice daily, each injected with 15 μl CORT solution) whereas those in replicate two were fed 4 mg of CORT per day (one mealworm per feed, twice daily each injected with 20 μl CORT solution).

### Judgment bias task

#### Task overview

The task comprised five stages: acclimatisation, pre-training 1, pre-training 2, discrimination training and cognitive bias testing. All trials began with a bird being placed in the start box and offered two mealworms (to ensure birds were currently motivated to eat mealworms) after which the door was opened to allow the bird access to the test arena. All trials ended with the bird being returned either to the start box to begin the next trial, or to its holding pen at the end of the session. An inter-trial interval of one minute was maintained throughout in order to clean the test arena and to set up the next trial. Behavioural testing took place between 0900 and 1300 each day.

#### Acclimatisation (day 4)

The aim of this stage was to teach the birds to enter the arena and locate and eat mealworms. Three petri dishes each containing 2 mealworms were distributed between the start box and the POS location in the test arena. The bird was allowed up to 2 mins to enter the arena and eat the mealworms. Each bird had four trials. Birds were paired during the first two trials to facilitate acclimatisation to the novel environment, but were alone on the third and fourth trial and in all subsequent training sessions. A bird was required to eat mealworms alone in order to progress to the next stage of training.

#### Pre-training 1 (day 5)

The aim of this stage was to teach the birds an association between paper cones and mealworms. Three petri dishes each containing a paper cone (made from a 3-cm diameter circle of paper cut to the centre and taped into a cone) lying on its side with 2 mealworms inside arranged such that the mealworms were visible from the start box were distributed between the start box and the POS location in the test arena. The bird was allowed up to 1 min to enter the arena and eat the mealworms. Each bird had five trials. A bird was required to successfully eat 20 mealworms on at least 4 trials to proceed to the next stage of training.

#### Pre-training 2 (day 6)

The aim of this stage was to teach the bird to displace a cone positioned in the rewarded location to access mealworms underneath. A petri dish containing two mealworms partially covered by a cone was placed at the POS location of the test arena. The bird was allowed up to 1 min to access and eat the mealworms. Each bird had five trials. A bird was required to successfully access the mealworms in at least three trials in order to proceed to the next stage of training.

#### Discrimination training (days 7–10)

The aim of this stage was to teach the birds that cones in the POS location were associated with mealworms but cones in the NEG location were associated with an aversive air puff. In this stage of training the cone in the NEG location was placed in a petri dish that had a hole drilled in the bottom so that an air puff could be delivered from beneath the cone if the bird displaced it. We predicted that learning would be exhibited by birds displaying longer latencies to approach and displace the cones in the NEG location compared with cones in the POS location. Each bird had twelve trials each day divided into two sessions of 6 trials. On day 7 all birds received the sequence POS-POS-NEG-NEG-POS-POS in their first session followed by a pseudorandom sequence of 3 POS and 3 NEG trials in their second session (where POS designates a cone with 2 mealworms at the POS location, and NEG a cone at the NEG location, the displacement of which was punished with a short air puff); the bird was allowed up to 2 min to displace the cone and, on POS trials, eat the mealworms. On days 8–10 there were again two sessions of 6 trials, and all trials were given in a pseudorandom sequence with the constraints that 6 POS and 6 NEG trials were presented in total with a maximum of two consecutive trials of the same type. The bird was allowed up to 1 min to displace a cone. The latency between the door of the start box being opened and the bird displacing the cone was recorded by an observer blind to the experimental treatment of the birds; trials in which the bird did not displace the cone were scored as the maximum possible latency of 60 s. To progress to judgment bias testing, a bird was required to show a significant difference in latency to displace POS and NEG cones in the pooled data from days 8–10 and, additionally in the data from just the final day of training (day 10).

#### Judgment bias testing (days 13–15)

The aim of this stage was to record how fast the birds approached and displaced cones in untrained, ambiguous locations (NNEG, MID and NPOS) compared to their latencies in the trained POS and NEG locations. Daily testing commenced 7 mins after consumption of the morning treated mealworm(s); this delay was chosen to ensure that CORT levels would have reached a maximum in the CORT-treated birds^34^. Birds received nine trials each day comprising three with the cone in the POS position (rewarded with 2 mealworms), three with the cone in the NEG position (punished with an air puff) and three with the cone in untrained, ambiguous, intermediate positions (NNEG, MID and NPOS; all unrewarded and unpunished). Each day, trials were delivered in three blocks of three trials each, with each block comprising one NEG, one POS and one ambiguous trial. The order of trials within each block was randomly chosen, as was the block in which each of the three ambiguous trials appeared. Birds were tested in staggered pairs (one CORT and one control), with the two birds alternating blocks until each had completed 12 trials. Between blocks, birds were placed in a holding pen identical to their home pen. All birds were tested for three consecutive days. Latencies were recorded as during discrimination training.

### Blood sampling and organ collection

Birds were weighed at the beginning and end of the treatment (days 11 and 17). At the end of day 17 a 0.5 ml sample of blood was taken from the inter-tarsal vein. Five drops of blood were put into an i-STAT cartridge (CG8^+^, Quality Clinical Reagent, Limited, York, UK) that was inserted into a blood gas analyser (Abaxis Vet Scan i-STAT®1 Analyser, Quality Clinical Reagent, Limited, York, UK) to obtain estimates of blood partial pressure of carbon dioxide (pCO_2_), pH, sodium ion content (Na^+^) and blood glucose. After blood sampling, birds were euthanized by cervical dislocation. A post mortem was undertaken to recover the liver, heart and spleen, all of which were weighed.

### Data Analysis

Raw data from the study are available as Supplementary Data S1 and S2. Data were analysed using the statistical computation package R^35^, and the R script that reproduces the analyses and figures reported in this paper is available in the Supplementary Methods. A criterion for significance of p < 0.05 was assumed unless otherwise stated.

In the analysis of the behavioural data from the judgment bias experiment our main dependent variable was the latency to displace the cone (this varied from 0 to a maximum possible of 60 s), and we used the latencies of birds in individual trials as the unit of analysis. To test whether individual birds showed significant differences in latency to POS and NEG following discrimination training and during judgment bias testing we used non-parametric Wilcoxon two-sample tests due to the relatively small number of data points and departures from normality in the data. To check for maintenance of the discrimination during cognitive bias testing we used a less conservative one-tailed test, because we had a clear *a priori* prediction about the direction of the difference expected, and we did not wish to exclude trained birds unnecessarily at this stage of the experiment. To model the cognitive bias test data we used general linear mixed models (GLMMs) implemented in the R package ‘nlme’^36^. Although latencies in the cognitive bias test trials were theoretically bounded between 0 and 60 s, inspection of residuals from the fitted models showed that assumptions of normality and homogeneity of variance were not violated, hence a Gaussian error structure was assumed. No adjustment was made for censoring^37^, since birds responded on or before the time limit of 60 s in the majority (76%) of the ambiguous test trials. GLMMs included a random intercept for bird, because repeated measures on individual birds were present. Model estimation was by maximum likelihood, and whether parameters differed significantly from zero was determined by testing the change in deviance when a given predictor was excluded from the model by comparing the likelihood ratio (LR) with the X^2^ distribution (all tests with 1 df). In the interests of keeping the statistical models as simple as possible, replicate was not included in the models because this variable was balanced in the experimental design: there were equal numbers of CORT and control birds from each replicate (4 in each treatment from replicate 1 and 3 in each treatment from replicate 2).

## Electronic supplementary material

Supplementary Methods

Supplementary Dataset 1

Supplementary Dataset 2
