# Supplementary material for: Elevated levels of the stress hormone, corticosterone, cause ‘pessimistic’ judgment bias in broiler chickens
Source: Sci Rep. 2017 Jul 31;7:6860. doi: 10.1038/s41598-017-07040-y (PMC5537245; doi:10.1038/s41598-017-07040-y)
Supplement: Supplementary file 1 — Supplementary Methods [file 41598_2017_7040_MOESM1_ESM.doc]

**Elevated levels of the stress hormone, corticosterone, cause ‘pessimistic’ judgment bias in broiler chickens**

Oluwaseun S. Iyasere^1, 2^, Andrew P. Beard^1^, Jonathan H. Guy^1, †^ and Melissa Bateson^3, †, *^.

^1^School of Agriculture, Food and Rural Development, Newcastle University, Newcastle upon Tyne, NE1 7RU, UK.

^2^Current address: Department of Animal Physiology, Federal University of Agriculture, Abeokuta, Ogun State, Nigeria, P.M.B 2240

^3^Institute of Neuroscience and Centre for Behaviour and Evolution, Newcastle University, Framlington Place, Newcastle upon Tyne, NE2 4HH, UK.

†Joint senior authors.

*Correspondence: melissa.bateson@ncl.ac.uk

**Supplementary methods**

# R data analysis script for Iyasere et al

# Melissa Bateson May 2017

# Data are read in from two files: one short and fat (by bird) and one long and thin (by trial)

library(reshape2)

library(nlme)

library(plyr)

library(ggplot2)

library(QuantPsyc) # to calculate standardised betas

library(car)

library(psych)

library(corrplot)

library(PerformanceAnalytics)

library(grid)

library(gridExtra)

library(AICcmodavg)

# read in the data

a = read.csv("Supplementary Data S1.csv")

d = read.csv("Supplementary Data S2.csv")

###### Analysis of training data ###########

xtabs(~d$Bird + d$Day)

train = d[d$Day<13,] # make a subset with just the training data

# Make a new data frame for the analysis of the training data

Bird = unique(train$Bird)

sum = data.frame(Bird)

sum$MWtestW8 = NA

sum$MWtestP8 = NA

sum$MWtestW9 = NA

sum$MWtestP9 = NA

sum$MWtestW10 = NA

sum$MWtestP10 = NA

sum$MWtestWall = NA

sum$MWtestPall = NA

# Conduct Mann-Whitney tests on each bird on each training day and save test stat and p-values

for(i in 1:length(Bird)) {

sub = train[train$Bird==sum$Bird[i],]

MWtest = wilcox.test(Latency ~ Valence, data=sub)

sum$MWtestPall[i] = MWtest$p.value

sum$MWtestWall[i] = MWtest$statistic

sub = train[train$Bird==sum$Bird[i]&train$Day==8,]

MWtest = wilcox.test(Latency ~ Valence, data=sub)

sum$MWtestP8[i] = MWtest$p.value

sum$MWtestW8[i] = MWtest$statistic

sub = train[train$Bird==sum$Bird[i]&train$Day==9,]

MWtest = wilcox.test(Latency ~ Valence, data=sub)

sum$MWtestP9[i] = MWtest$p.value

sum$MWtestW9[i] = MWtest$statistic

sub = train[train$Bird==sum$Bird[i]&train$Day==10,]

MWtest = wilcox.test(Latency ~ Valence, data=sub)

sum$MWtestP10[i] = MWtest$p.value

sum$MWtestW10[i] = MWtest$statistic

}

all = merge(a, sum, by = c('Bird'), all = TRUE)

# Calculate the number of air puffs received during discrimination phase for the successfully trained birds

train2= train[train$Bird!=81 & train$Bird!=110,] # eliminate two unsuccessful birds (81 and 110)

train2$Flip[train2$Latency==120 & train2$Day==7]=0

train2$Flip[train2$Latency<120 & train2$Day==7]=1

train2$Flip[train2$Latency==60 & train2$Day>7]=0

train2$Flip[train2$Latency<60 & train2$Day>7]=1

# calculate the number of air puffs received by each bird during training

tf3 = ddply(train2, .(Bird, Valence), summarise, Flip=sum(Flip, na.rm=TRUE))

tf4 = ddply(tf3, .(Valence), summarise, No.flipped=mean(Flip), sd=sd(Flip),n=length(Flip))

# latencies

tf1 = ddply(train2, .(Bird, Day, Valence), summarise, Latency=mean(Latency, na.rm=TRUE))

tf2 = ddply(tf1, .(Day, Valence), summarise, lat=mean(Latency), sd=sd(Latency),n=length(Latency))

##### Analysis of anatomical data ########

xtabs(~a$Treatment) # n= 23 birds: 11 control and 12 cort birds

describeBy(a, group = a$Treatment)

# Variables are: WeightChange, Spleen, Liver and Heart (all in grammes)

a$WeightChange = a$EndWeight-a$StartWeight

# Simple t-tests for variables where correction for body weight is not necessary

t.test(WeightChange ~ Treatment, data = a, na.action = na.omit)

t.test(pH ~ Treatment, data = a, na.action = na.omit)

wilcox.test(pH ~ Treatment, data = a, na.action = na.omit)

t.test(PCO2 ~ Treatment, data = a, na.action = na.omit)

wilcox.test(PCO2 ~ Treatment, data = a, na.action = na.omit)

t.test(Na ~ Treatment, data = a, na.action = na.omit)

wilcox.test(Na ~ Treatment, data = a, na.action = na.omit)

t.test(Glucose ~ Treatment, data = a, na.action = na.omit)

wilcox.test(Glucose ~ Treatment, data = a, na.action = na.omit)

# ANCOVAs for the organ weights where we need to correct for body weight

a2 = aov(Spleen ~ Treatment + EndWeight, data = a, na.action = na.omit)

Anova(a2, type="III")

a3 = aov(Liver ~ EndWeight + Treatment, data = a, na.action = na.omit)

Anova(a3, type="III")

a4 = aov(Heart ~ EndWeight + Treatment, data = a, na.action = na.omit)

Anova(a4, type="III")

# Calculate critical p-value for Bonferroni correction:

0.05/8 # gives 0.00625 for alpha

# Calculate relative spleen and liver weights

all$RelSpleen = all$Spleen/all$EndWeight

all$RelLiver = all$Liver/all$EndWeight

#### Analysis of cognitive bias test data

d = d[d$Day>=13,] # make a subset with just the test data

# calculate average speeds to attack POS and NEG

pos = d[d$Valence=="POS1"|d$Valence=="POS2"|d$Valence=="POS3",]

posm = ddply(pos, .(Bird), summarise, POSmean=mean(Latency, na.rm=TRUE),POSse=sd(Latency,na.rm=TRUE)/3)

neg = d[d$Valence=="NEG1"|d$Valence=="NEG2"|d$Valence=="NEG3",]

negm = ddply(neg, .(Bird), summarise, NEGmean=mean(Latency, na.rm=TRUE), NEGse=sd(Latency,na.rm=TRUE)/3)

# do the same for NNEG

nneg = d[d$Valence=="NNEG",]

nnegm = ddply(nneg, .(Bird), summarise, NNEGmean=mean(Latency, na.rm=TRUE), NNEGse=sd(Latency,na.rm=TRUE)/sqrt(3))

# and for all the intermediates too

amb = d[d$Valence=="NNEG"|d$Valence=="MID"|d$Valence=="NPOS",]

ambm = ddply(amb, .(Bird), summarise, AMBmean=mean(Latency, na.rm=TRUE), AMBse=sd(Latency,na.rm=TRUE)/sqrt(9))

# merge these averages into the main data frame

all = merge(all, posm, by = "Bird", all = TRUE)

all = merge(all, negm, by = "Bird", all = TRUE)

all = merge(all, ambm, by = "Bird", all = TRUE)

all = merge(all, nnegm, by = "Bird", all = TRUE)

all$MeanSpeed = (all$POSmean +all$NEGmean)/2

# recode the valences as numbers:

d$ValenceN[d$Valence == "POS1"|d$Valence == "POS2"|d$Valence == "POS3"] = 5

d$ValenceN[d$Valence == "NEG1"|d$Valence == "NEG2"|d$Valence == "NEG3"] = 1

d$ValenceN[d$Valence == "NPOS"] = 4

d$ValenceN[d$Valence == "MID"] = 3

d$ValenceN[d$Valence == "NNEG"] = 2

# Do birds retain the POS NEG discrimination in the test trials?

# Calculate whether the latencies to POS and NEG are significantly different in test sessions

# this is done with both Mann-Whitney U tests and T-tests and create new columns

p = d[d$ValenceN == 1|d$ValenceN == 5,] # make a subset of the POS and NeG trials only

# Bird 81 (cort gp) needs to be dropped due to lack of discrimination.

d=d[d$Bird!=81,]

d = droplevels(d)

p=p[p$Bird!=81,]

p=droplevels(p)

trained = all[is.na(all$MeanSpeed)==FALSE&all$Bird!=81,]

trained = droplevels(trained)

trained$TtestT = NA

trained$TtestP = NA

trained$MWtestW = NA

trained$MWtestP = NA

for(i in 1:nrow(trained)) {

sub = p[p$Bird==trained$Bird[i],]

MWtest = wilcox.test(Latency ~ as.factor(ValenceN), data=sub, alternative = "greater")

trained$MWtestP[i] = MWtest$p.value

trained$MWtestW[i] = MWtest$statistic

ttest = t.test(Latency ~ ValenceN, data=sub)

trained$TtestP[i] = ttest$p.value

trained$TtestT[i] = ttest$statistic

}

# Pessimism index

trained$PessNNEG = (trained$NNEGmean-trained$POSmean)/(trained$NEGmean-trained$POSmean)

trained$PessAMB = (trained$AMBmean-trained$POSmean)/(trained$NEGmean-trained$POSmean)

# Censoring?

t = d[d$Valence == "NPOS"|d$Valence == "MID"|d$Valence == "NNEG",] # subset of ambiguous trials

c = t[t$Latency == 60,] # subset censored

propc = 1-(length(c$Latency)/length(t$Latency)) # proportion of ambiguous trials not censored

propc

# PLot a graph of all the individual birds' cog bias data

fg = ggplot(data=d) +

geom_point(aes(x=as.factor(ValenceN), y=Latency, colour = "Red"), size=1.8) +

scale_x_discrete(limits=c("1", "2", "3", "4", "5"), labels=c("NEG", "NN", "M", "NP", "POS")) +

stat_summary(aes(x=ValenceN, y=Latency), geom="line", fun.y=mean, colour = "Black") +

theme_bw() +

theme(panel.grid.major = element_blank(), panel.grid.minor = element_blank()) +

theme(axis.text.x = element_text(angle = 45, vjust = 1, hjust=1)) +

scale_colour_discrete(guide=FALSE) +

scale_shape_discrete(guide=FALSE) +

facet_grid(Treatment ~ Bird) +

xlab("Valence") +

ylab("Latency (s)")

print(fg)

# Analysis of POS and NEG in test trials

t1 = lme(Latency ~ as.factor(ValenceN) + Treatment + as.factor(ValenceN)*Treatment, random = ~1|Bird, data = p, na.action = na.omit, method = "ML")

plot(t1)

hist(resid(t1))

summary(t1)

t2 = lme(Latency ~ as.factor(ValenceN) + Treatment, random = ~1|Bird, data = p, na.action = na.omit, method = "ML")

summary(t2)

anova(t1,t2) # tests the interaction between valence and treatment

t3 = lme(Latency ~ as.factor(ValenceN), random = ~1|Bird, data = p, na.action = na.omit, method = "ML")

anova(t2,t3) # tests Treatment

t4 = lme(Latency ~ Treatment, random = ~1|Bird, data = p, na.action = na.omit, method = "ML")

anova(t2,t4) # tests Valence

# Note that treatment is not significant in this analysis (p = 0.18)

# Is there a significant difference between birds?

# use the gls command to compare the model with and without the random effect of bird

b2 = gls(Latency ~ as.factor(ValenceN) + Treatment + as.factor(ValenceN)*Treatment, data = p, na.action = na.omit, method = "ML")

summary(b2)

anova(t1,b2) # test whether the random effect of bird contributes to the fit of the model

# Analysis of ambiguous test trials

# Merge in the Mean speed data for each bird

t = merge(t,all, by = c("Bird","Treatment"))

# Does treatment predict how birds respond to ambiguous stimuli?

m1 = lme(Latency ~ MeanSpeed + ValenceN + Treatment + ValenceN*Treatment, random = ~1|Bird, data = t, na.action = na.omit, method = "ML")

plot(m1)

hist(resid(m1))

summary(m1)

aicmod1=AICc(m1)

m2 = lme(Latency ~ MeanSpeed + ValenceN + Treatment, random = ~1|Bird, data = t, na.action = na.omit, method = "ML")

anova(m1,m2) # tests the interaction between valence and treatment

m3 = lme(Latency ~ MeanSpeed + ValenceN, random = ~1|Bird, data = t, na.action = na.omit, method = "ML")

anova(m2,m3) # tests treatment

m4 = lme(Latency ~ MeanSpeed + Treatment, random = ~1|Bird, data = t, na.action = na.omit, method = "ML")

anova(m2,m4) # tests valence

m5 = lme(Latency ~ ValenceN + Treatment + ValenceN*Treatment, random = ~1|Bird, data = t, na.action = na.omit, method = "ML")

anova(m1,m5) # tests mean speed

# Does adding replicate make any difference?

xtabs(~trained$Replicate + trained$Treatment)

# numbers of birds in each treatment the same in each replicate therefore replicate cannot bias results

# Does adding test day make any difference?

m1 = lme(Latency ~ Day + MeanSpeed + ValenceN + Treatment + ValenceN*Treatment, random = ~1|Bird, data = t, na.action = na.omit, method = "ML")

plot(m1)

hist(resid(m1))

summary(m1)

m2 = lme(Latency ~ Day + MeanSpeed + ValenceN + Treatment, random = ~1|Bird, data = t, na.action = na.omit, method = "ML")

anova(m1,m2) # tests the interaction between valence and treatment

m3 = lme(Latency ~ Day + MeanSpeed + ValenceN, random = ~1|Bird, data = t, na.action = na.omit, method = "ML")

anova(m2,m3) # tests treatment

m4 = lme(Latency ~ Day + MeanSpeed + Treatment, random = ~1|Bird, data = t, na.action = na.omit, method = "ML")

anova(m2,m4) # tests valence

m5 = lme(Latency ~ Day + ValenceN + Treatment + ValenceN*Treatment, random = ~1|Bird, data = t, na.action = na.omit, method = "ML")

anova(m1,m5) # tests mean speed

m6 = lme(Latency ~ MeanSpeed + ValenceN + Treatment + ValenceN*Treatment, random = ~1|Bird, data = t, na.action = na.omit, method = "ML")

anova(m1,m6) # tests TestDay.

# Draw a graph of main cog bias result

fig = ddply(d, .(Bird, Treatment, ValenceN), summarise, Latency=mean(Latency, na.rm=TRUE))

figd = ddply(fig, .(Treatment, ValenceN), summarise, lat=mean(Latency), sd=sd(Latency),n=length(Latency))

figd$se = figd$sd/sqrt(figd$n)

fig2 = ggplot(figd, aes(x=ValenceN, y=lat, fill = Treatment)) +

geom_line(position=position_dodge(0.2)) +

geom_errorbar(aes(ymin=figd$lat-figd$se, ymax=figd$lat+figd$se), width=0.2, position=position_dodge(0.2)) +

geom_point(shape=22, size=4, position=position_dodge(0.2)) +

scale_fill_manual(values=c("black", "white"), labels=c("Control","CORT")) +

scale_x_continuous(breaks=c(1,2,3,4,5),labels=c("NEG","NNEG","MID","NPOS","POS")) +

xlab("Valence") +

ylab("Latency (s)") +

theme_bw() +

theme(panel.grid.major = element_blank()) + # switch off major gridlines

theme(panel.grid.minor = element_blank()) +

theme(legend.position=c(1,1), legend.justification=c(1,1)) +

theme(legend.background=element_rect(colour="grey50"))

fig2

png('Figure2.png', width = 6, height = 6, units = 'in', res = 1200)

print(fig2)

dev.off()

######## Correlations between anatomical data and cog bias data ###############

# use trained = data frame of just birds with cog bias data

colnames(trained)

# made a data frame containing the measures we wabt to correlate

attach(trained)

dat = data.frame(RelSpleen, RelLiver, MeanSpeed, PessNNEG)

detach(trained)

dat2 = rename(dat, c("RelSpleen"="Spleen", "RelLiver"="Liver", "MeanSpeed"="Speed","PessNNEG"="PI NNEG"))

matrix = cor(dat2) # correlation matrix of these data

matrix

# Make a nice figure

corrplot(matrix, type = "lower", diag = FALSE, tl.srt=45, tl.col="black",method = "ellipse",addCoef.col="black")

png('Figure3.png', width = 6, height = 6, units = 'in', res = 1200)

corrplot(matrix, type = "lower", diag = FALSE, tl.srt=45, tl.col="black",method = "ellipse",addCoef.col="black")

dev.off()

# Does relative spleen weight predict how birds respond to ambiguous stimuli?

m1 = lme(Latency ~ MeanSpeed + ValenceN + RelSpleen + ValenceN*RelSpleen, random = ~1|Bird, data = t, na.action = na.omit, method = "ML")

plot(m1)

hist(resid(m1))

summary(m1)

aicmod2=AICc(m1)

m2 = lme(Latency ~ MeanSpeed + ValenceN + RelSpleen, random = ~1|Bird, data = t, na.action = na.omit, method = "ML")

anova(m1,m2) # tests the interaction between valence and spleen

m3 = lme(Latency ~ MeanSpeed + ValenceN, random = ~1|Bird, data = t, na.action = na.omit, method = "ML")

anova(m2,m3) # tests main effect of spleen

m4 = lme(Latency ~ MeanSpeed + RelSpleen, random = ~1|Bird, data = t, na.action = na.omit, method = "ML")

anova(m2,m4) # tests valence

m5 = lme(Latency ~ ValenceN + RelSpleen + ValenceN*RelSpleen, random = ~1|Bird, data = t, na.action = na.omit, method = "ML")

anova(m1,m5) # tests mean speed

# Draw a graph of main cog bias result split by spleen

# Need to do a median split of the data on RelSpleen

trained$RelSpleen.cat[trained$RelSpleen<=median(trained$RelSpleen)]="Relatively small spleens"

trained$RelSpleen.cat[trained$RelSpleen>median(trained$RelSpleen)]="Relatively large spleens"

fin = merge(trained, d, by = "Bird")

fig = ddply(fin, .(Bird, RelSpleen.cat, ValenceN), summarise, Latency=mean(Latency, na.rm=TRUE))

figd = ddply(fig, .(RelSpleen.cat, ValenceN), summarise, lat=mean(Latency), sd=sd(Latency),n=length(Latency))

figd$se = figd$sd/sqrt(figd$n)

fig4 = ggplot(figd, aes(x=ValenceN, y=lat, fill = RelSpleen.cat)) +

geom_line(position=position_dodge(0.2)) +

geom_errorbar(aes(ymin=figd$lat-figd$se, ymax=figd$lat+figd$se), width=0.2, position=position_dodge(0.2)) +

geom_point(shape=22, size=4, position=position_dodge(0.2)) +

scale_fill_manual(values=c("black", "white"), labels=c("Large","Small")) +

scale_x_continuous(breaks=c(1,2,3,4,5),labels=c("NEG","NNEG","MID","NPOS","POS")) +

xlab("Valence") +

ylab("Latency (s)") +

labs(fill = "Relative spleen weight") +

theme_bw() +

theme(panel.grid.major = element_blank()) +

theme(panel.grid.minor = element_blank()) +

theme(legend.position=c(1,1), legend.justification=c(1,1)) +

theme(legend.background=element_rect(colour="grey50"))

fig4

png('Figure4.png', width = 6, height = 6, units = 'in', res = 1200)

print(fig4)

dev.off()
